# Early prediction of sepsis associated encephalopathy in elderly ICU patients using machine learning models: a retrospective study based on the MIMIC-IV database

**DOI:** 10.3389/fcimb.2025.1545979

**Published:** 2025-04-17

**Authors:** Yupeng Han, Xiyuan Xie, Jiapeng Qiu, Yijie Tang, Zhiwei Song, Wangyu Li, Xiaodan Wu

**Affiliations:** ^1^ Department of Anesthesiology, Shengli Clinical Medical College of Fujian Medical University, Fujian Provincial Hospital, Fuzhou University Affiliated Provincial Hospital, Fuzhou, Fujian, China; ^2^ Department of Neurology, Shengli Clinical Medical College of Fujian Medical University, Fujian Provincial Hospital, Fuzhou University Affiliated Provincial Hospital, Fuzhou, Fujian, China; ^3^ Department of Pain Management, The First Affiliated Hospital of Fujian Medical University, Fuzhou, Fujian, China; ^4^ Fujian Provincial Key Laboratory of Critical care Medicine, Fujian Provincial Hospital, Fuzhou University Affiliated Provincial Hospital, Fuzhou, Fujian, China

**Keywords:** machine learning, early prediction, sepsis associated encephalopathy, elderly, MIMIC-IV

## Abstract

**Background:**

Sepsis associated encephalopathy (SAE) is prevalent among elderly patients in the ICU and significantly affects patient prognosis. Due to the symptom similarity with other neurological disorders and the absence of specific biomarkers, early clinical diagnosis remains challenging. This study aimed to develop a predictive model for SAE in elderly ICU patients.

**Methods:**

The data of elderly sepsis patients were extracted from the MIMIC IV database (version 3.1) and divided into training and test sets in a 7:3 ratio. Feature variables were selected using the LASSO-Boruta combined algorithm, and five machine learning (ML) models, including Extreme Gradient Boosting (XGBoost), Categorical Boosting (CatBoost),Light Gradient Boosting Machine(LGBM), Multilayer Perceptron (MLP), and Support Vector Machines (SVM), were subsequently developed using these variables. A comprehensive set of performance metrics was used to assess the predictive accuracy, calibration, and clinical applicability of these models. For the machine learning model with the best performance, we employed the SHapley Additive Explanations(SHAP) method to visualize the model.

**Results:**

Based on strict inclusion and exclusion criteria, a total of 3,156 elderly sepsis patients were enrolled in the study, with an SAE incidence rate of 48.7%. The mortality rate of elderly sepsis patients who developed SAE was significantly higher than that of patients in the non-SAE group (28.78% vs. 12.59%, *P* < 0.001). A total of 18 feature variables were selected for the construction of the ML model using the LASSO-Boruta combined algorithm. Compared to the other four models and traditional scoring systems, the XGBoost model demonstrated the best overall predictive performance, with Area Under the Curve(AUC)=0.898, accuracy=0.830, recall=0.819, F1-Score=0.820, specificity=0.840, and Precision=0.821. Furthermore, the results from the Decision Curve Analysis (DCA) and calibration curves demonstrated that the XGBoost model has significant clinical value and stable predictive performance. The ten-fold cross-validation method further confirmed the robustness and generalizability of the model. In addition, we simplified the model based on the SHAP feature importance ranking, and the results indicated that the simplified XGBoost model retains excellent predictive ability (AUC=0.858).

**Conclusions:**

The XGBoost model effectively predicts SAE in elderly ICU patients and may serve as a reliable tool for clinicians to identify high-risk patients.

## 1 Introduction

Sepsis-associated encephalopathy (SAE) is a severe neurological syndrome characterized by sepsis-induced acute diffuse cerebral dysfunction (Bleck et al., 1993; Gofton and Young, 2012; Heming et al., 2017). Early clinical features of SAE include impaired consciousness, cognitive decline, altered mental status, and, in severe cases, coma, which may lead to long-term neurocognitive deficits and a high mortality rate (Sonneville et al., 2023). The onset of SAE involves complex mechanisms, including abnormal neuroinflammatory and immune responses, blood-brain barrier damage, and microcirculatory disorders (Ren et al., 2020; Hong et al., 2023). Previous studies have reported a significant correlation between the occurrence and severity of SAE and patient prognosis, with even mild alterations in consciousness (GCS score of 13 or 14) significantly increasing the risk of death (Sonneville et al., 2017).

With the aging population, elderly patients with septic encephalopathy have garnered increased attention (Manabe and Heneka, 2022). Existing research suggests that the incidence of sepsis and SAE is notably higher among elderly ICU patients, with age being a significant high-risk factor affecting short-term survival in patients who have developed SAE (Ljungström et al., 2019; Chen et al., 2020; Zhang et al., 2024). This reciprocal causality adds significant complexity to the clinical treatment and management of elderly patients with SAE. Additionally, it has been reported that even among elderly patients who survive sepsis, many develop long-term cognitive deficits during recovery (Iwashyna et al., 2010; Muzambi et al., 2021). Although early recognition of high-risk patients and prompt interventions can substantially enhance prognosis, factors such as atypical clinical symptoms and comorbidities associated with elderly SAE complicate the identification and early prediction of risk factors. Therefore, an in-depth analysis of the clinical characteristics of this patient group is essential.

In recent years, machine learning (ML) techniques have demonstrated significant potential for predicting, diagnosing, and assessing the risk of sepsis and its associated complications (Yue et al., 2022; Li et al., 2024; Lin et al., 2024; Prithula et al., 2024). ML is a computational method that builds data models by analyzing large and multidimensional datasets to make predictions. It works by using historical data (such as patient age, medical history, and lab results) to identify variables that may significantly impact the onset and progression of diseases, rather than relying on predefined programming rules. As a result, ML can provide more accurate diagnosis and prognosis assessments compared to traditional predictive models, helping clinicians identify early risks and intervention opportunities. In diagnosing and prognosticating SAE, researchers have utilized machine learning models to conduct comprehensive analyses of clinical, laboratory, and demographic characteristics, providing more personalized prediction tools for clinical risk screening of SAE patients (Lu et al., 2022; Peng et al., 2022). However, evidence supporting the superiority of ML models in the early prediction of SAE occurrence in elderly sepsis patients remains limited. This study aims to develop several ML models for the early prediction of SAE occurrence in elderly sepsis patients in ICU and identify the model with the highest predictive performance.

## 2 Materials and methods

### 2.1 Data source

The data used to construct the model were obtained from the single-center database of the Medical Information Marketplace in Intensive Care IV (MIMIC-IV, version v3.1). This database contains clinical information on all patient hospitalizations admitted to the ICU at Beth Israel Deaconess Medical Center from 2008 to 2022, including demographics, length of hospitalization, ICU admissions and discharges, vital signs, laboratory data, medications, and nursing care records (Johnson et al., 2024, Johnson et al., 2023). To request access to the database, the author of this study (YP.H.) completed the Collaborative Institutional Training Initiative (CITI) program exam and received a certificate (ID: 59425375). Because the MIMIC database is de-identified and does not contain private patient information, the Institutional Review Board at Beth Israel Deaconess Medical Center waived the requirement for informed consent.

### 2.2 Participants

The study included patients who fulfilled the following criteria: (1) age ≥65 years; (2) met Sepsis-3.0 criteria and were admitted to the ICU; (3) no diagnosis of sepsis-associated encephalopathy at the time of ICU admission; (4) length of stay in the ICU ≥24 hours; (5) for patients with multiple ICU admissions, data were collected only for the first admission. In addition, we excluded the following categories of patients: (1) patients with combined brain parenchymal injury (cerebral infarction, cerebral hemorrhage, traumatic brain injury) and other cerebrovascular diseases; (2) patients with mental disorders or dementia; (3) patients with alcohol or drug addiction; (4) patients with hepatic or renal encephalopathy and suspected metabolic encephalopathy (e.g., hyponatremia [<120 mmol/L], hyperglycemia [>180 mg/dL], or hypoglycemia [<54 mg/dL]); and (5) patients with missing delirium assessment or missing data >30%.

**Figure 1:**
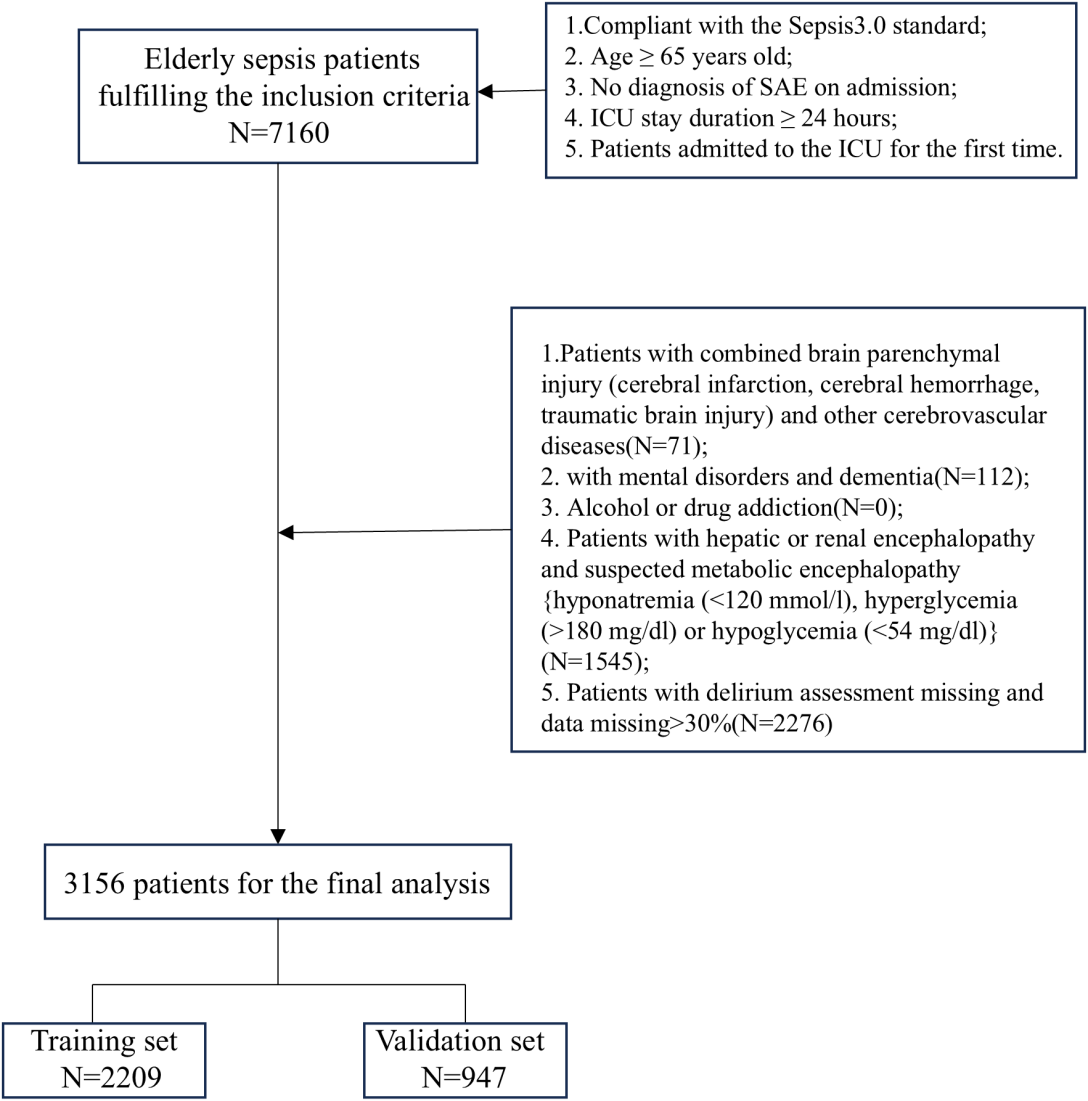
Flow chart of the patient selection.

The primary outcome of the study was the occurrence of SAE in elderly sepsis patients after the first day of ICU admission. The diagnostic criteria for SAE included meeting the SEPSIS 3.0 criteria, along with a GCS score <15 or a positive ICU delirium assessment (Hong et al., 2023; Sonneville et al., 2023; Kurtz et al., 2024). The Delirium Assessment Scale for the ICU (CAM-ICU) was used to assess delirium in ICU patients. Based on the aforementioned diagnostic criteria and to ensure baseline consistency among study participants, we excluded patients who were diagnosed with altered consciousness (GCS < 15) or delirium within the first 24 hours of admission. Additionally, because this was a hypothesis-driven epidemiological study, no attempt was made to estimate the sample size, and all eligible elderly septic patients in the dataset were included to maximize statistical power. The flow chart of patient selection is shown in **Figure 1**.

### 2.3 Data extraction

Structured Query Language in PostgreSQL was used to extract case information from the database for patients who met the inclusion criteria. The extracted data for this study included the following variables: (1) Demographic information: age, gender, and race; (2) Comorbidities: hypertension (HTN), type 1 diabetes (T1DM), type 2 diabetes (T2DM), hyperlipidemia (HLD), coronary artery disease (CAD), chronic obstructive pulmonary disease (COPD), chronic kidney disease (CKD), and Charlson Comorbidity Index; (3) Vital signs and disease scores at the time of admission: heart rate (HR), respiratory rate (RR), non-invasive mean arterial pressure (MAP), oxygen saturation (SpO2), and disease scores including Sequential Organ Failure Assessment(SOFA), Oxygenation Assessment Index Score (OASIS), Simplified Acute Physiology Score II (SAPS II), and Acute Physiology Score III(APS III); (4) Laboratory indices within 24 hours of admission: white blood cell count (WBC), platelet count (PLT), red blood cell count (RBC), red cell distribution width (RDW), hemoglobin, hematocrit, PCO_2_, pH, glucose, potassium, sodium, chloride, anion gap, lactate, prothrombin time (PT), international normalized ratio (INR), creatinine, and blood urea nitrogen (BUN); (5) Prognostic data included therapeutic measures during hospitalization (e.g., Ventilation, continuous renal replacement therapy (CRRT), vasopressors, sedatives and analgesics, Glucocorticoids drugs), length of ICU stay, and 28-day mortality. Additionally, to minimize the impact of missing data on model construction, variables with less than 20% missing data were interpolated using the KNNImputer (KNN) method, while those with more than 20% missing were discarded. The advantage of the KNNImputer method lies in its ability to fill missing values by leveraging the similarity between data points, thereby preserving the inherent structure and relationships of the data. Compared to traditional methods like mean or median imputation, KNNImputer handles complex, nonlinear data distributions more effectively, particularly in cases with multiple missing features. Furthermore, KNNImputer does not require specific distribution assumptions, offering greater flexibility and enhancing the accuracy and stability of predictive models (Akter et al., 2024; Guan et al., 2024).

### 2.4 Feature selection

After including patients based on strict adherence to the inclusion and exclusion criteria, we split the dataset into training and test sets in a 7:3 ratio using the Bootstrap sampling technique. The Bootstrap method is a robust and flexible tool for statistical inference and model evaluation. Bootstrap generates a large volume of sample data through repeated sampling, which helps balance differences in sample distributions and enables the full modeling of the sampling distributions for both the model and control groups, thus facilitating the assessment of differences or relationships between groups. Additionally, the final feature variables used in the logistic regression and machine learning models were selected using LASSO regression and Boruta methods based on the validation set (Li et al., 2024). The LASSO method selects features and reduces dimensionality by shrinking coefficients, retaining features with larger contributions and eliminating redundant ones. The Boruta algorithm identifies the most important features by comparing the Z-value of each feature to that of the “shadow features.” The common feature variables identified by both methods were selected as the final feature set for the model. This approach enhances model accuracy while reducing the risk of overfitting and excluding irrelevant predictors. Considering that variables within the first 24 hours of admission better reflect the patient’s initial health status and disease severity, which are crucial for predicting early risks and clinical outcomes, while daily treatment measures during hospitalization are more influenced by changes in the patient’s condition and are less effective in predicting early SAE occurrence, this study included only monitoring data from the first 24 hours of admission in the variable selection.

### 2.5 Construction and validation of machine learning models

After feature selection, five machine learning models were employed for constructing and validating the diagnostic model using identical training and validation sets. These models include three ensemble algorithms—Categorical Boosting (CatBoost), Extreme Gradient Boosting (XGBoost), and Light Gradient Boosting Machine (LGBM)—and two conventional base algorithms: Multilayer Perceptron (MLP) and Support Vector Machine (SVM). The models were trained on the training set, and the test set was used for model validation. We evaluated the performance of the ML prediction models using metrics such as Area Under the Curve (AUC), specificity, recall, F1 score, and accuracy to identify the best diagnostic model. Additionally, calibration curves, precision-recall (PR) curves, and Decision Curve Analysis (DCA) were used to evaluate the calibration and clinical applicability of the ML models. Finally, for the best-performing diagnostic models, we revalidated their generalization ability and robustness using 10-fold cross-validation to prevent overfitting.

### 2.6 Model interpretation and feature importance

SHapley Additive Explanations(SHAP) is a game-theory-based model interpretation method used to explain the output of machine learning models. SHAP interprets the impact of each feature on the final prediction by considering all possible combinations and orders of features and calculating each feature’s contribution in those combinations (Wang et al., 2021). The SHAP feature importance ranking and SHAP bees plot show each feature’s contribution to the final prediction, while the SHAP force diagram offers an intuitive visualization of how different features influence individual predictions. In our work, we use the aforementioned SHAP methods to visualize the best-performing ML models in terms of efficacy, thereby enhancing their interpretability.

### 2.7 Comparison of the optimal model with traditional scoring systems

To assess whether predictive models outperform traditional methods in early prediction of SAE in elderly ICU patients, We evaluated the best models against traditional scoring systems using the same dataset.

### 2.8 Simplification of the best machine learning prediction model

In this study, we aim to simplify the model with the highest predictive efficacy based on the SHAP feature importance ranking results. The simplified model not only reduces the complexity of clinical decision-making but also enables clinicians to quickly assess the patient’s condition in daily practice, thereby enhancing the efficiency and accuracy of clinical decision-making.

### 2.9 Statistical analysis

Data analysis was performed using DecisionLinnc1.0 software, a platform that integrates multiple programming language environments for data processing, analysis, and machine learning model construction through a visual interface (DecisionLinnc Core Team, 2023). The Kolmogorov-Smirnov test was used for continuous variables. As these variables were non-normally distributed, they are presented as median (interquartile range), with differences between groups assessed using the Mann-Whitney U test. Categorical variables are presented as percentages (%), and group differences were compared with the Pearson chi-square test, with p-values < 0.05 considered statistically significant.

## 3 Results

### 3.1 Comparison of clinical information of patients

A total of 3,156 elderly sepsis patients were included in the study based on rigorous inclusion and exclusion criteria, with 1,620 patients in the non-SAE group and 1,536 in the SAE group, resulting in an SAE incidence rate of 48.7%.The overall missing data situation is shown in **Supplementary Table 1**. Patients in the SAE group had a higher median age compared to those in the non-SAE group (*P* < 0.05). In terms of comorbidities, the SAE group exhibited a higher Charlson Comorbidity Index, a greater proportion of patients with comorbid COPD and AKI, and a lower proportion of those with hyperlipidemia and ischemic heart disease (IHD) (*P* < 0.05). Concerning vital signs upon admission and disease severity scores, the SAE group had significantly higher median values for MAP, RR, HR, and disease scores (SOFA, OASIS, SAPS II, and APS III), while their median SpO^2^ was lower compared to the non-SAE group (*P* < 0.05). Above results are presented in detail in **Table 1**.

**Table 1:** Comparison of baseline characteristics in the NonSAE and SAE groups.

	Overall	NonSAE	SAE	*P*
	n=3156	n=1620	n=1536	
Age(year)	76 (70,83)	75 (70,82)	76 (70,83)	0.015
Gender				0.064
Female, n(%)	1329 (42.11)	656 (40.49)	673 (43.82)	
Male, n(%)	1827 (57.89)	964 (59.51)	863 (56.18)	
Race				0.002
White, n(%)	2069 (65.55)	1122 (69.30)	947 (61.65)	
Black, n(%)	190 (6.02)	82 (5.06)	108 (7.03)	
Asian, n(%)	74 (2.34)	43 (2.65)	31 (2.02)	
Hispanic, n(%)	62 (1.96)	34 (2.10)	28 (1.82)	
Others, n(%)	761 (24.11)	339 (20.93)	422 (27.47)	
Complications				
Hypertension, n(%)	1492 (47.28)	787 (48.58)	705 (45.90)	0.141
T1 Diabetes, n(%)	17 (0.54)	10 (0.62)	7 (0.46)	0.707
T2 Diabetes, n(%)	822 (26.05)	430 (26.54)	392 (25.52)	0.54
Hyperlipidemia, n(%)	1630 (51.65)	903 (55.74)	727 (47.33)	<0.001
Icoronary heart disease, n(%)	1560 (49.43)	853 (52.65)	707 (46.03)	<0.001
COPD, n(%)	605 (19.17)	269 (16.60)	336 (21.88)	<0.001
Chronic kidney disease, n(%)	752 (23.83)	351 (21.67)	401 (26.11)	0.004
Charlson	6 (4,8)	5 (4,7)	6 (4,8)	<0.001
Admission vital signs				
MAP (mmHg)	75 (66,87)	74 (65,84)	77 (67,90)	<0.001
Respiratory rate (beats/minute)	18 (15,22)	17 (14,21)	19 (16,23)	<0.001
Heart rate (beats/minute)	83 (73,96)	81 (74,93)	85 (73,99)	0.01
SPO_2_(%)	99 (96,100)	99 (96,100)	98 (95,100)	<0.001
System score				
SOFA	5 (3,7)	4 (3,6)	5 (3,8)	<0.001
OASIS	32 (27,38)	30 (25,35)	34 (29,39)	<0.001
SapsII	39 (32,46)	36 (31,43)	41 (34,49)	<0.001
ApsIII	41 (31,53)	38 (30,48)	44 (34,56)	<0.001

COPD, Chronic obstructive pulmonary disease; SOFA, Sequential Organ Failure Assessment; OASIS, Outcome and Assessment Information Set; SAPS II, Simplified Acute Physiology Score II; Charlson, Charlson Comorbidity Index; APSS III, Acute Physiology Score System III.

Laboratory data comparison (**Table 2**) showed that WBC, PLT, RBC, RDW, Hemoglobin, Hematocrit, PCO_2_, Glucose, Sodium, Anion gap, Creatinine, and BUN levels were higher in the elderly SAE group compared to the non-SAE group (*P* < 0.05). In contrast, PCO2, pH, and chloride levels were lower in the SAE group (*P* < 0.05). No significant differences were found in coagulation function indices, including PTT, PT, INR, potassium, and lactate (*P* > 0.05).

**Table 2:** Comparison of laboratory examination data between two groups of patients on admission.

	Overall	NonSAE	SAE	*P*
	n=3156	n=1620	n=1536	
WBC(10^9^/L)	11.3 (8.075,16)	11.2 (7.9,15.8)	11.4 (8.1,16.1)	0.006
PLT(10^9^/L)	162 (119,230)	152 (114.75,213)	176 (127,244.25)	<0.001
RBC (10^9^/L)	3.37 (2.87,3.89)	3.3 (2.86,3.79)	3.44 (2.89,4)	<0.001
RDW(%)	14.5 (13.4,15.9)	14.2 (13.3,15.6)	14.7 (13.6,16.1)	<0.001
Hemoglobin(g/dL)	10.1 (8.6,11.7)	10 (8.6,11.4)	10.3 (8.7,11.908)	<0.001
Hematocrit(%)	31 (26.5,35.8)	30.3 (26.2,34.6)	31.7 (27,36.7)	<0.001
PCO_2_(mmHg)	40.9 (36,46)	40.25 (37,45)	41 (36,47)	<0.001
PO_2_(mmHg)	149.2(68,275)	191(82,311)	115.54(61,212)	<0.001
Glucose (mg/dl)	122 (104,143)	120 (103,139)	125 (104,146.25)	<0.001
Potassium(mmol/L)	4.1 (3.8,4.6)	4.2 (3.8,4.6)	4.1 (3.7,4.6)	0.112
Sodium(mmol/L)	139 (136,141)	139 (136,141)	139 (136,142)	0.004
Chloride(mmol/L)	106 (101,109)	106 (102,110)	105 (101,109)	<0.001
Aniongap(mmol/l)	14 (11,16)	13 (11,16)	14 (12,17)	<0.001
Lactate (mmol/l)	1.885 (1.3,2.6)	1.96 (1.4,2.6)	1.8 (1.2,2.6)	0.184
PTT (seconds)	31.42 (27.9,37.6)	31.28 (27.8,37.2)	31.7 (28,38.3)	0.367
PT (seconds)	14.6(12.9,17.025)	14.9 (13.2,17.178)	14.3 (12.7,17)	0.15
INR	1.3 (1.2,1.6)	1.4 (1.2,1.6)	1.3 (1.2,1.6)	0.146
Creatinine (mg/dl)	1 (0.8,1.6)	1 (0.8,1.5)	1.1 (0.8,1.7)	0.003
BUN(mg/dl)	22 (15,34)	20 (15,30)	24 (16,39)	<0.001

WBC, White Blood Cell; PLT, Platelet; RBC, Red Blood Cell; RDW, Red Cell Distribution Width; PCO_2_, Partial Pressure of Carbon Dioxide; PO_2_, Partial Pressure of Oxygen; PH, Potential of Hydrogen; APTT, Activated Partial Thromboplastin Time; PT, Prothrombin Time; INR, International Normalized Ratio; BUN, Blood Urea Nitrogen.

Regarding treatment, a higher proportion of patients in the SAE group received vasopressin, sedatives, analgesics, and corticosteroids (*P* < 0.05). The SAE group also had a greater proportion of patients requiring ventilation and CRRT (*P* < 0.05). Moreover, the ICU stay duration, and 28-day mortality rate were significantly higher in the SAE group compared to the non-SAE group (*P* < 0.05). The results are presented in **Table 3**.

**Table 3:** Comparison of treatment and prognosis between two groups of patients.

	Overall	NonSAE	SAE	*P*
	n=3156	n=1620	n=1536	
Vasopressin, n (%)	2296 (72.75)	1125 (69.44)	1171 (76.24)	<0.001
Sedative and analgesic drugs, n (%)	2328 (73.76)	1021 (63.02)	1307 (85.09)	<0.001
Glucocorticoids drugs, n (%)	784 (24.84)	344 (21.23)	440 (28.65)	<0.001
ventilation, n (%)	2833 (89.77)	1397 (86.23)	1436 (93.49)	<0.001
CRRT, n (%)	209 (6.62)	54 (3.33)	155 (10.09)	<0.001
ICU Duration (day)	3.11 (1.79-6.13)	2.01 (1.32-3.08)	5.53(3.29-9.85)	<0.001
ICU 28 day mortality, n (%)	646 (20.47)	204 (12.59)	442 (28.78)	<0.001

### 3.2 Feature selection

**Figure 2:**
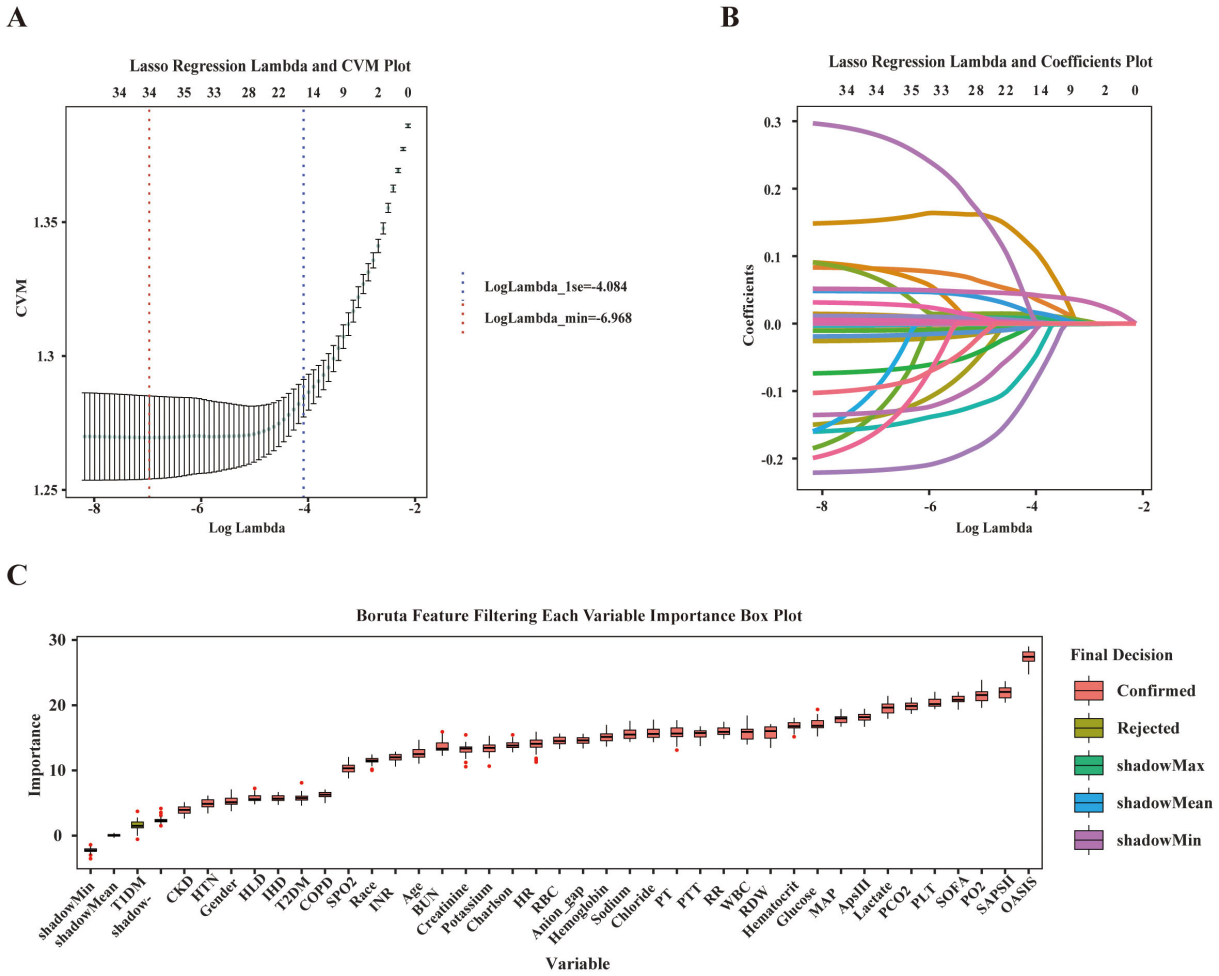
Feature selection. **(A)** The relationship between Lambda (regularization parameter) and CVM (mean cross validation error) in Lasso regression; **(B)** Lasso regression Lambda and Coefficients Plot. **(C)** Feature selection based on Boruta principle.

We sequentially used Lasso regression and the Boruta method to identify relevant features from the training set. In LASSO regression, the variable coefficients are presented in **Figure 2A**, and the relationship between the regularization parameter (λ) and the mean cross-validation error (CVM) is depicted in **Figure 2B**. These results indicate that λ = 0.0009 (i.e., Logλ-min = -6.968) is the optimal value for achieving the model’s highest efficacy. The 18 variables identified in the LASSO regression as strongly associated with the occurrence of SAE in elderly sepsis patients included MAP, RR, HTN, COPD, CKD, SOFA, OASIS, SAPS II, Charlson, WBC, sodium, PLT, hematocrit, glucose, anion gap, PCO_2_, PTT, and BUN. The regression coefficients for the variables in the LASSO regression are provided in **Supplementary Table 2**. Subsequently, the Boruta method identified only T1DM as an irrelevant variable (**Figure 2C**). Ultimately, the 18 variables identified above were included in the subsequent analysis.

### 3.3 Model performance comparisons

**Figure 3:**
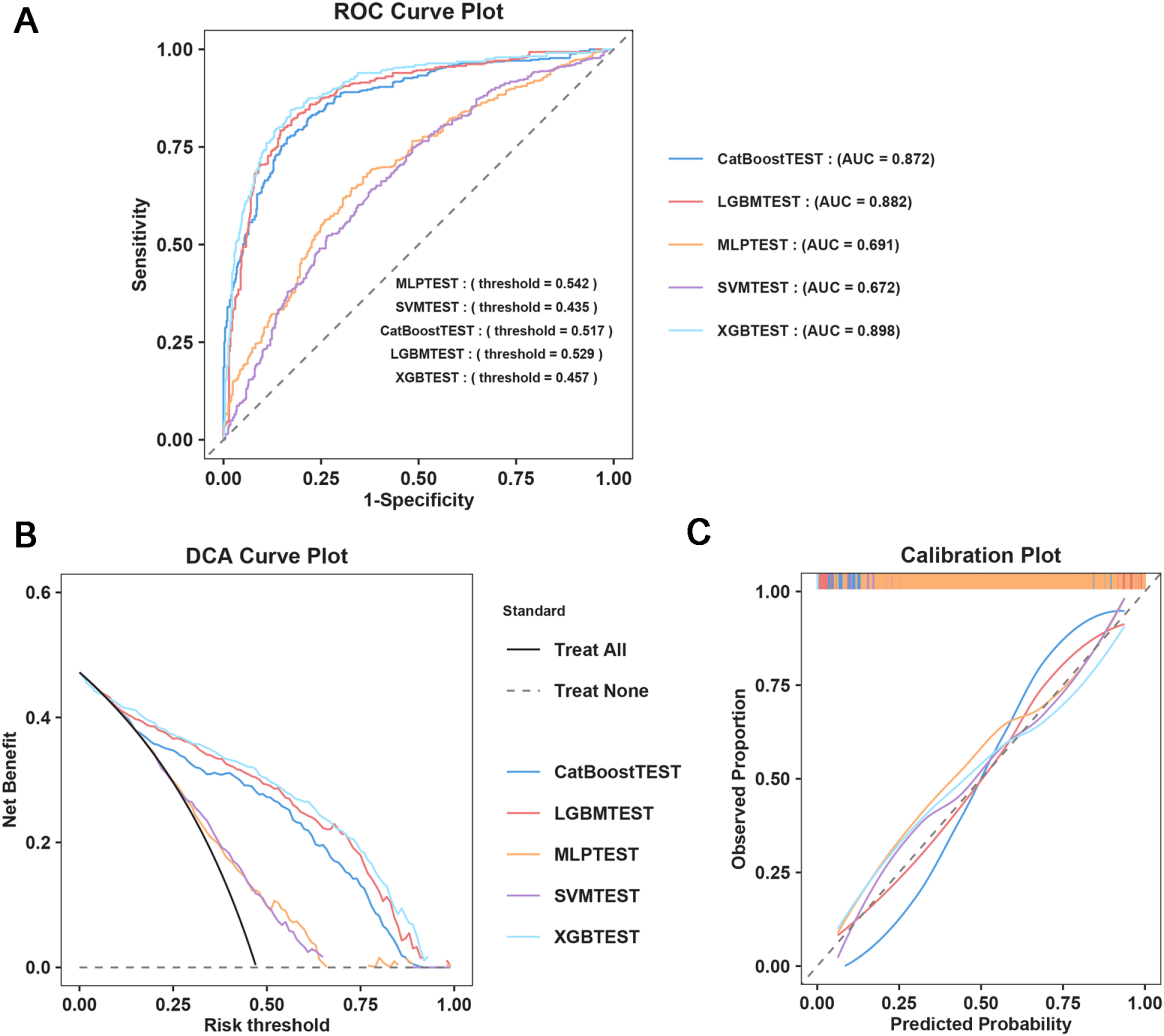
Machine learning model construction and diagnostic energy efficiency evaluation. **(A)** ROC Curve Plot; **(B)** DCA Curve Plot; **(C)** Calibration Plot.

Five ML models were developed to assess the risk of SAE in elderly sepsis patients in our study. The ROC curves demonstrated that the three models from the integrated algorithm (XGBoost, LGBM, and CatBoost) exhibited good predictive performance for new SAEs in elderly sepsis patients, outperforming the MLP and SVM models based on the common algorithm (**Figure 3A**). Among these models, the XGBoost model (AUC=0.898) demonstrated the best performance, followed by LGBM (AUC = 0.882), CatBoost (AUC = 0.872), MLP (AUC = 0.691), and SVM (AUC = 0.672). In terms of clinical applicability, the three models from the integrated algorithm demonstrated consistent net benefits across various threshold probabilities, with the XGBoost model providing the greatest net benefit (**Figure 3B**), demonstrating the significant clinical value of our developed ML model. Additionally, the calibration curves confirmed the stability of the results for each model (**Figure 3C**). We also examined the detailed performance metrics of the aforementioned ML models (shown in **Table 4**), and the results indicated that the XGBoost model outperformed the other four models.

**Table 4:** Performances of the machine learning models for predicting SAE in elderly ICU patients.

Model	Accuracy	AUC	Recall	F1-Score	Precision	Specificity
XGBoost	0.830	0.898	0.819	0.820	0.821	0.840
LGBM	0.820	0.882	0.803	0.809	0.814	0.836
CatBoost	0.800	0.872	0.781	0.787	0.793	0.818
SVM	0.628	0.672	0.642	0.620	0.599	0.616
MLP	0.626	0.691	0.720	0.645	0.584	0.542

**Figure 4:**
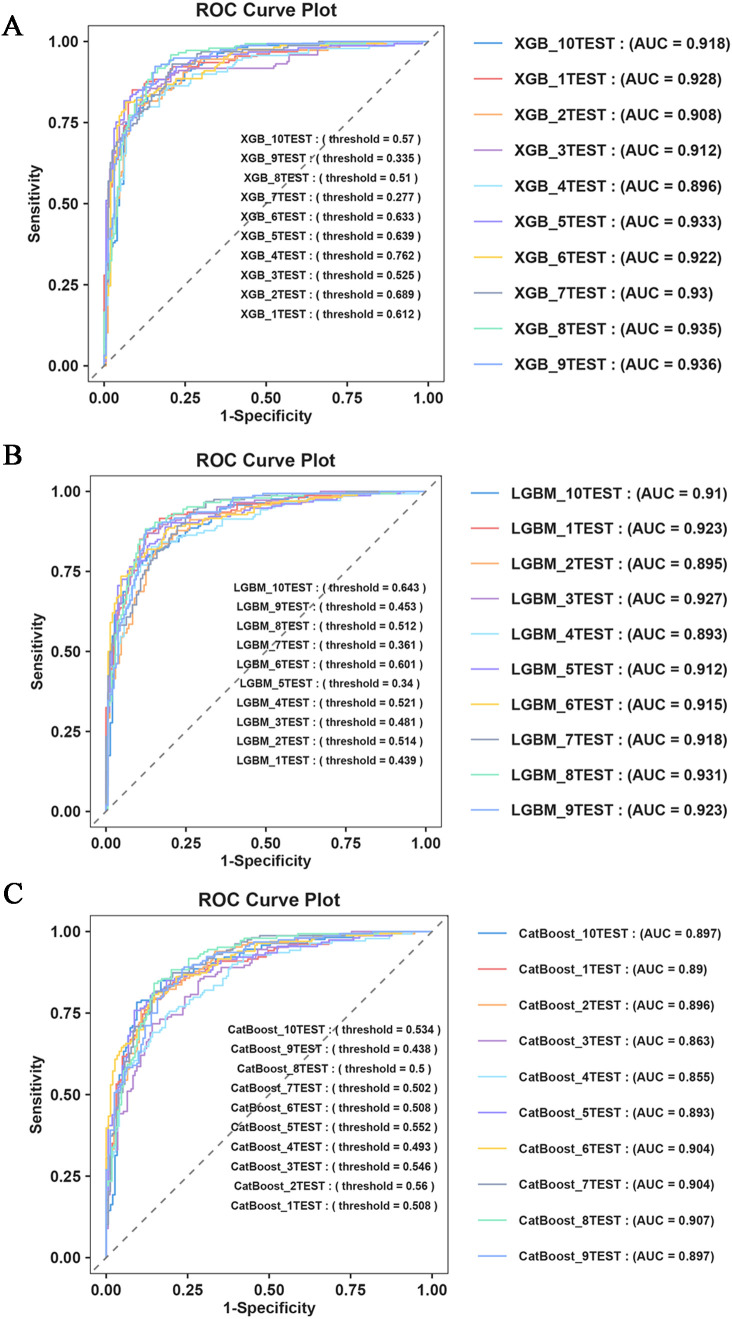
Ten fold cross validation evaluation. **(A)** ROC Curve Plot of XGBoost; **(B)** ROC Curve Plot of LGBM; **(C)** ROC Curve Plot of CatBoost.

Furthermore, to assess the robustness and generalization ability of the three models—XGBoost, LGBM, and CatBoost—we re-examined the predictive efficacy of the models using ten-fold cross-validation. The detailed performance metrics (**Table 5**) and ROC curve results (**Figure 4**) confirm that our constructed model demonstrates good robustness and generalization ability, with no signs of overfitting or underfitting. Based on these results, the XGBoost model was identified as the best-performing model in this study.

**Table 5:** Ten fold cross validation evaluation of three ensemble algorithm ML models.

Model	Accuracy	AUC	Recall	F1-Score	Precision	Specificity
XGBoost	0.852	0.922	0.847	0.847	0.847	0.856
LGBM	0.844	0.915	0.836	0.838	0.842	0.852
Catboost	0.816	0.891	0.852	0.799	0.817	0.833

### 3.4 Comparison of the optimal model with traditional scoring systems

We compared the XGBoost model with traditional scoring systems using the same dataset. Our results revealed that traditional scoring systems (SOFA, OASIS, SAPS II, APS III) exhibited poor predictive efficacy, with all AUC values falling below 0.7 (**Table 6**).

**Table 6:** Comparison of the optimal model with traditional scoring systems.

Variable	Accuracy	AUC	Recall	Precision	Specificity
SOFA	0.568	0.563	0.368	0.627	0.775
OASIS	0.614	0.651	0.506	0.654	0.725
SapsII	0.603	0.618	0.572	0.617	0.635
APSIII	0.578	0.593	0.641	0.575	0.513
XGBoost	0.830	0.898	0.819	0.820	0.821

### 3.5 Model visualization based on SHAP principle

**Figure 5:**
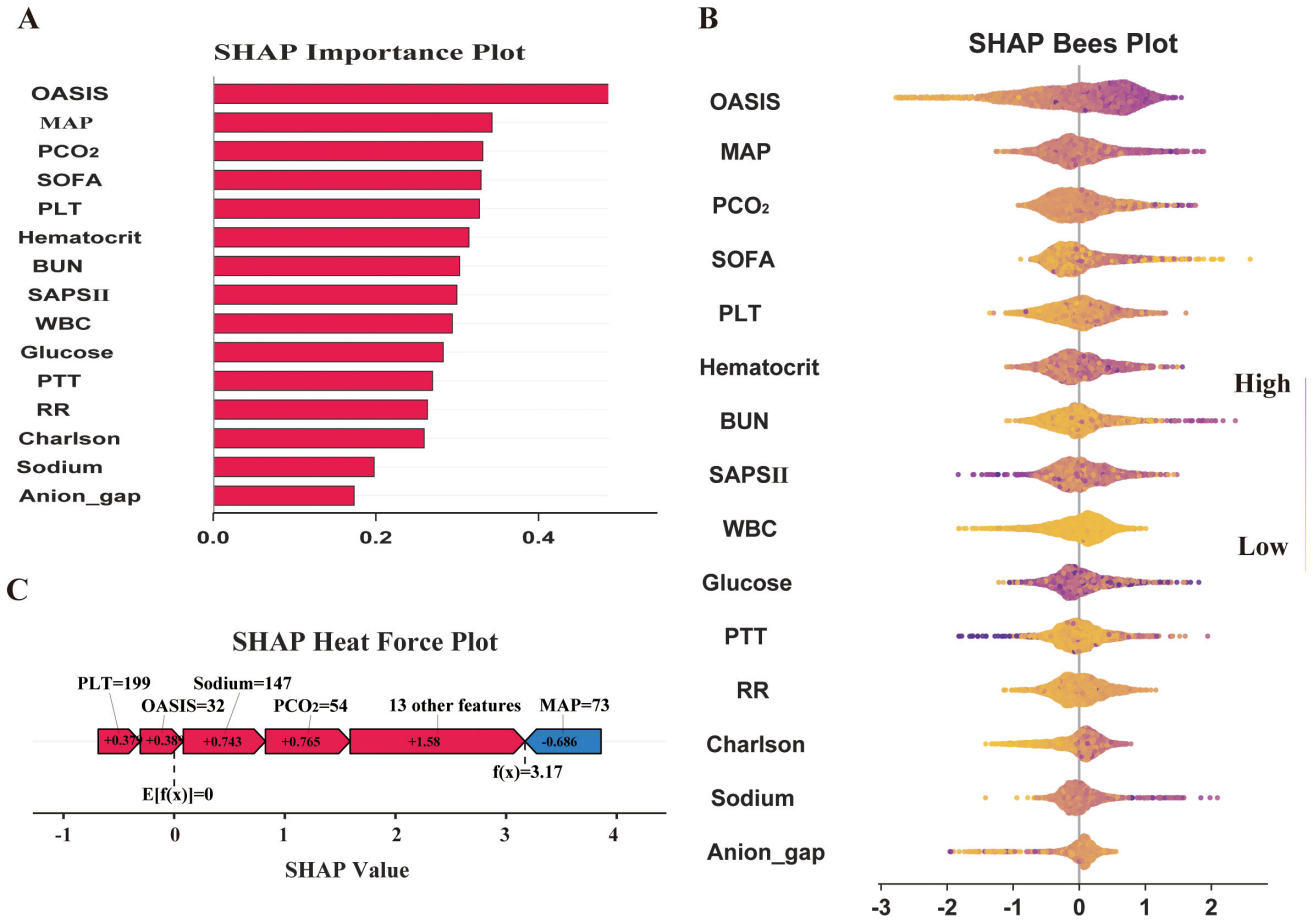
Model interpretation and feature importance. **(A)** SHAP Importance Plot; **(B)** SHAP Bees Plot; **(C)** SHAP Heat Force Plot.

**Figure 6:**
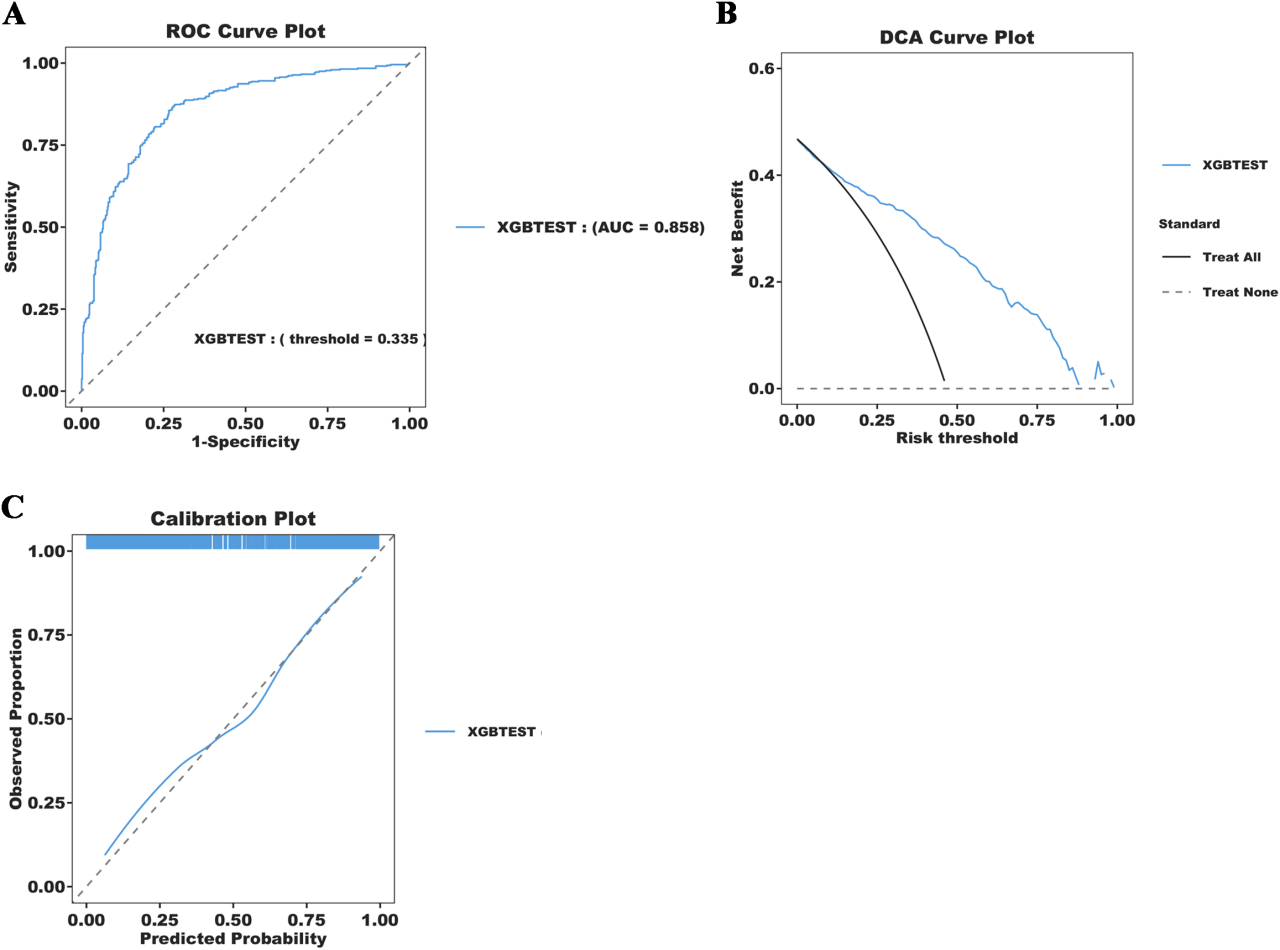
Simplification of the XGBoost Prediction Model construction and diagnostic energy efficiency evaluation. **(A)** ROC Curve Plot; **(B)** DCA Curve Plot;**(C)** Calibration Plot.

We focus on visualizing the contribution of feature variables in the XGBoost model using the SHAP (Shapley Additive Explanations) principle. The SHAP feature importance ranking and swarm plots display the relative contribution of each feature to the model’s global prediction outcomes, while the SHAP force plots illustrate the contribution of these factors for a specific individual. The SHAP-based feature importance ranking plot (**Figure 5A**) and the swarm plot (**Figure 5B**) show the global contribution of feature variables incorporated in the XGBoost model, with the horizontal axis representing the SHAP values, indicating each feature’s contribution to the model’s predicted outcomes. The vertical axis ranks the features based on the impact of their cumulative SHAP values. Our results demonstrate that OASIS, MAP, PCO2, SOFA, and PLT are the five most important features influencing new-onset SAEs in elderly patients with sepsis. The SHAP force plot (**Figure 5C**) highlights the direction and magnitude of each feature’s influence on the prediction for a specific elderly patient with SAE, based on the XGBoost model for this particular outcome. In this visualization, red indicators represent a positive impact, while blue indicators denote a negative impact. Notably, our results show that for this particular patient, the critical values influencing the likelihood of an SAE are PLT = 199, Oasis = 32, Sodium = 147, PCO_2_ = 54, and MAP = 73. In addition, we further simplified the XGBoost predictive model by utilizing these five metrics. The results indicate that the simplified model maintains excellent predictive ability (AUC=0.858), and the DCA curve, along with the calibration curve, validate the reliability of the findings (**Figure 6**).

## 3 Discussion

In this study, we constructed a prediction model based on ML models for the risk of SAE in elderly sepsis patients admitted to ICU. We identified 18 clinical variables through the use of LASSO combined with the Boruta method and constructed six machine learning models using these variables. Subsequent results demonstrated that the XGBoost algorithm model exhibited the best predictive performance among all ML models and provided substantial clinical utility, as confirmed by the DCA curve analysis. The ten-fold cross-validation results provided additional confirmation of the stability and clinical utility of the XGBoost algorithm model for predicting SAE diagnosis in elderly patients.

SAE is recognized as the most common encephalopathy in the ICU, with incidence rates varying across studies and populations, typically ranging from 10% to 50% globally (Gofton and Young, 2012; Sonneville et al., 2017, Sonneville et al., 2023). In our study, the incidence was 48.7%, which is relatively high. Compared to elderly sepsis patients without neurological disorders, those with SAE had significantly longer ICU stays and higher 28-day mortality risk, highlighting the severity and complexity of sepsis in the elderly. Several previous studies have identified age as an important risk factor in the onset and progression of SAE (Ljungström et al., 2019; Chen et al., 2020; Zhang et al., 2024). Therefore, Prompt detection of high-risk patients at risk of developing SAE is crucial for elderly sepsis patients admitted to ICU. Unfortunately, although existing guidelines for sepsis management emphasize early recognition and prompt treatment of sepsis (Evans et al., 2021), they mainly focus on signs of sepsis, source of infection control, and organ function support, and fail to provide an in-depth analysis of elderly septic patients, including those with SAE (Gamboa-Antiñolo, 2021). Traditional sepsis scoring systems such as SOFA, Oasis, Saps II, and Aps III are widely used in the critical care assessment of sepsis (Qiu et al., 2023; Fan and Ma, 2024), but our results demonstrated limited effectiveness in identifying elderly SAE, with AUC values consistently below 0.7. In fact, these traditional scoring systems primarily focus on physiological parameters and organ failure. Therefore, despite their validity in assessing the severity and short-term prognosis of sepsis, they are not well-suited for diagnosing and predicting sepsis-associated encephalopathy.

In recent years, ML has demonstrated great potential in the diagnosis and prognosis of sepsis and its associated complications. By analyzing comprehensive clinical data, ML models are able to identify potential risk factors for the development of sepsis and predict the progression of the disease, with primary applications in early prediction, risk assessment, and the development of personalized treatment strategies (Fleuren et al., 2020; Komorowski et al., 2022; Upadhyaya et al., 2025). The introduction of machine learning has improved the accuracy and efficiency of sepsis management and offered robust support for clinical decision-making. For example, Li et al. constructed an ML-based model from the data of 1,663 patients receiving RRT in the MIMIC database and found that the LR model exhibited outstanding performance in predicting the risk of clinical prognosis in patients with sepsis-associated acute kidney injury undergoing RRT (Li et al., 2024). Notably, ML methods have also demonstrated significant potential in the diagnosis and prognosis of the SAE, and several researchers have built models for early diagnosis and short-term prognostic risk assessment of high-risk patients with SAE using ML approaches (Ge et al., 2022; Lu et al., 2022; Peng et al., 2022; Guo et al., 2023). However, there remains a gap in machine learning models specifically for predicting SAE risk in elderly sepsis patients.

Among the five ML prediction models developed in this study, the three integration algorithm-based models—XGBoost, LGBM, and CatBoost—outperform the MLP and SVM models that utilize standard algorithms. This finding suggests that the integrated learning approach performs more effectively for this type of prediction task, likely due to its ability to combine multiple weak learners and enhance the generalization and accuracy of the model. Our results demonstrate that the geriatric SAE prediction model based on the XGBoost algorithm exhibits the best overall performance, surpassing the other four ML models and the traditional ICU condition scoring tool. This outcome is consistent with findings from previous studies (Lu et al., 2022; Zhang et al., 2023). XGBoost is an efficient, gradient boosting algorithm widely used for classification, regression, and ranking tasks. It has the advantage of combining multiple weak predictive models to generate accurate predictions. Due to its superior comprehensive performance, XGBoost ML has garnered increasing attention for predicting adverse clinical outcomes (Yue et al., 2022; Guan et al., 2024).

SHAP is a method used to explain the degree of contribution of feature variables in ML models and provides a clearer visual interpretation of model predictions. The global analysis utilizing SHAP identifies OASIS, MAP, PCO2, SOFA, and platelets as the top five factors influencing the occurrence of SAE in elderly sepsis patients. OASIS and SOFA are commonly employed in ICUs to assess patient condition (He et al., 2022; Fan and Ma, 2024), with higher scores typically indicating more severe disease states. This suggests that the patient’s physiological systems are under greater stress, leading to a significantly increased risk of SAE. Elevated MAP generally reflects higher blood pressure, which may impact cerebral blood flow, causing endothelial damage in cerebral vessels and exacerbating neurological symptoms or cerebral complications (Slessarev et al., 2020; Wang et al., 2022). This, in turn, increases the risk of SAEs in elderly patients with sepsis. Our modeling results also indicated that elevated blood indicators, such as platelets and PCO2, were associated with the occurrence of SAEs. Platelets, key cells in blood coagulation, are often activated in septic conditions, promoting thrombosis. When platelet counts are elevated, they not only raise the risk of thrombosis but also contribute to SAE by aggravating microvascular damage and promoting an inflammatory response, which can lead to organ failure (Fodil and Zafrani, 2022; Leung and Middleton, 2024). Finally, elevated PCO_2_ may be linked to brain tissue hypoxia and inadequate cerebral perfusion during sepsis. Sepsis-induced metabolic disturbances lead to carbon dioxide accumulation, raising PCO_2_ levels, which may worsen the mismatch in cerebral blood flow regulation (Carr et al., 2023; Caldwell et al., 2024), thereby impairing neurological function and the normal metabolism of brain cells. In summary, the combination of multiple physiological indicators and pathological states in elderly septic patients significantly increases the risk of SAE. Meanwhile, we further simplified the model using the above five indicators. The results indicate that the simplified model retains excellent predictive ability (AUC=0.858). The simplified model enables clinicians to quickly access and evaluate these key indicators, thereby improving prediction efficiency. These findings also underscore the significance of machine learning models in developing disease prediction systems.

This study has several limitations. First, patients with SAE often require sedation to control agitation, reduce metabolic demand, or improve ventilation tolerance (Helbing et al., 2018); however, sedation can obscure symptoms of delirium and cognitive impairment, potentially distorting assessment scores. The patient consciousness assessment data in this study were derived from electronic medical records available in the MIMIC database; however, they lacked key information, such as the sedation-to-assessment interval and the state of recovery of consciousness, which may have led to biased results. Second, the retrospective nature of this study, based on a single database, may restrict the applicability and generalizability of the findings. Future studies should incorporate multicenter ICU data and conduct prospective investigations to stratify patient populations more effectively (e.g., considering the timing of SAE onset and the type and timing of sedative medication use), allowing for a more comprehensive evaluation and refinement of the model. Moreover, this retrospective study is based on the MIMIC database, which primarily includes ICU patients from the United States. Its demographic composition may not fully represent global populations, limiting the generalizability of our findings. Ethnic differences can influence SAE presentation, treatment response, and outcomes (Haddad et al., 2020), while disparities in healthcare access and comorbidities may affect the external validity of our model. Future studies should validate these findings in diverse populations and assess whether race-specific model adjustments enhance performance across demographic groups. Finally, although the SHAP method was used to visually illustrate the high-risk factors for SAE occurrence in elderly patients, aiding physicians in identifying and understanding the most relevant clinical characteristics, further optimization of the model is needed. Future efforts should focus on optimizing the model, developing user-friendly interfaces (e.g., a mobile application or web tool), and exploring its integration with electronic medical record systems. This would enable clinicians to input patient data and retrieve predictive results without requiring programming expertise, thereby facilitating clinical decision-making.

## Data Availability

The original contributions presented in the study are included in the article/**Supplementary Material**. Further inquiries can be directed to the corresponding author.

## References

[B1] Akter S. Simul Hasan Talukder M. Mondal S. K. Aljaidi M. Bin Sulaiman R. Alshammari A. A. (2024). Brain tumor classification utilizing pixel distribution and spatial dependencies higher-order statistical measurements through explainable ML models. Sci. Rep. 14, 25800. doi: 10.1038/s41598-024-74731-8 39468107 PMC11519933

[B2] Bleck T. P. Smith M. C. Pierre-Louis S. J. Jares J. J. Murray J. Hansen C. A. (1993). Neurologic complications of critical medical illnesses. Crit. Care Med. 21, 98–103. doi: 10.1097/00003246-199301000-00019 8420739

[B3] Caldwell H. G. Hoiland R. L. Bain A. R. Howe C. A. Carr J. Gibbons T. D.. (2024). Evidence for direct CO(2) -mediated alterations in cerebral oxidative metabolism in humans. Acta physiologica (Oxford England) 240, e14197. doi: 10.1111/apha.14197 38958262

[B4] Carr J. Day T. A. Ainslie P. N. Hoiland R. L. (2023). The jugular venous-to-arterial PCO2 difference during rebreathing and end-tidal forcing: Relationship with cerebral perfusion. J. Physiol. (Lond.) 601, 4251–4262. doi: 10.1113/JP284449 37635691

[B5] Chen J. Shi X. Diao M. Jin G. Zhu Y. Hu W.. (2020). A retrospective study of sepsis-associated encephalopathy: epidemiology, clinical features and adverse outcomes. BMC Emerg Med. 20, 77. doi: 10.1186/s12873-020-00374-3 33023479 PMC7539509

[B6] DecisionLinnc Core Team (2023). DecisionLinnc. 1.0. Available online at: https://www.statsape.com/ (Accessed October 10, 2024).

[B7] Evans L. Rhodes A. Alhazzani W. Antonelli M. Coopersmith C. M. French C.. (2021). Surviving sepsis campaign: international guidelines for management of sepsis and septic shock 2021. Intensive Care Med. 47, 1181–1247. doi: 10.1007/s00134-021-06506-y 34599691 PMC8486643

[B8] Fan S. Ma J. (2024). The value of five scoring systems in predicting the prognosis of patients with sepsis-associated acute respiratory failure. Sci. Rep. 14, 4760. doi: 10.1038/s41598-024-55257-5 38413621 PMC10899590

[B9] Fleuren L. M. Klausch T. Zwager C. L. Schoonmade L. J. Guo T. Roggeveen L. F.. (2020). Machine learning for the prediction of sepsis: a systematic review and meta-analysis of diagnostic test accuracy. Intensive Care Med. 46, 383–400. doi: 10.1007/s00134-019-05872-y 31965266 PMC7067741

[B10] Fodil S. Zafrani L. (2022). Severe thrombotic thrombocytopenic purpura (TTP) with organ failure in critically ill patients. J. Clin. Med. 11, 1103. doi: 10.3390/jcm11041103 35207375 PMC8874413

[B11] Gamboa-Antiñolo F. M. (2021). Prognostic tools for elderly patients with sepsis: in search of new predictive models. Intern Emerg Med. 16, 1027–1030. doi: 10.1007/s11739-021-02729-5 33847904

[B12] Ge C. Deng F. Chen W. Ye Z. Zhang L. Ai Y.. (2022). Machine learning for early prediction of sepsis-associated acute brain injury. Front. Med. (Lausanne) 9. doi: 10.3389/fmed.2022.962027 PMC9575145 36262275

[B13] Gofton T. E. Young G. B. (2012). Sepsis-associated encephalopathy. Nat. Rev. Neurol. 8, 557–566. doi: 10.1038/nrneurol.2012.183 22986430

[B14] Guan C. Gong A. Zhao Y. Yin C. Geng L. Liu L.. (2024). Interpretabl e machine learning model for new-onset atrial fibrillation prediction in critically ill patients: a multi-center study. Crit. Care (London England) 28, 349. doi: 10.1186/s13054-024-05138-0 PMC11523862 39473013

[B15] Guo J. Cheng H. Wang Z. Qiao M. Li J. Lyu J. (2023). Factor analysis based on SHapley Additive exPlanations for sepsis-associated encephalopathy in ICU mortality prediction using XGBoost - a retrospective study based on two large database. Front. Neurol. 14. doi: 10.3389/fneur.2023.1290117 PMC10755941 38162445

[B16] Haddad D. N. Mart M. F. Wang L. Lindsell C. J. Raman R. Nordness M. F.. (2020). Socioeconomic factors and intensive care unit-related cognitive impairment. Ann. Surg. 272, 596–602. doi: 10.1097/SLA.0000000000004377 32932314 PMC7739516

[B17] He Y. Xu J. Shang X. Fang X. Gao C. Sun D.. (2022). Clinical characteristics and risk factors associated with ICU-acquired infections in sepsis: A retrospective cohort study. Front. Cell Infect. Microbiol 12. doi: 10.3389/fcimb.2022.962470 PMC9366915 35967847

[B18] Helbing D. L. Böhm L. Witte O. W. (2018). Sepsis-associated encephalopathy. CMAJ: Can. Med. Assoc. J. = J. l’Association medicale Can. 190, E1083. doi: 10.1503/cmaj.180454 PMC6131085 30201616

[B19] Heming N. Mazeraud A. Verdonk F. Bozza F. A. Chrétien F. Sharshar T. (2017). Neuroanatomy of sepsis-associated encephalopathy. Crit. Care (London England) 21, 65. doi: 10.1186/s13054-017-1643-z PMC5360026 28320461

[B20] Hong Y. Chen P. Gao J. Lin Y. Chen L. Shang X. (2023). Sepsis-associated encephalopathy: From pathophysiology to clinical management. Int. Immunopharmacol. 124, 110800. doi: 10.1016/j.intimp.2023.110800 37619410

[B21] Iwashyna T. J. Ely E. W. Smith D. M. Langa K. M. (2010). Long-term cognitive impairment and functional disability among survivors of severe sepsis. JAMA 304, 1787–1794. doi: 10.1001/jama.2010.1553 20978258 PMC3345288

[B22] Johnson A. Bulgarelli L. Pollard T. Gow B. Moody B. Horng S.. (2024). MIMIC-IV (version 3.1). PhysioNet. doi: 10.13026/kpb9-mt58

[B23] Johnson A. Bulgarelli L. Shen L. Gayles A. Shammout A. Horng S.. (2023). MIMIC-IV, a freely accessible electronic health record dataset. Sci. Data 10, 1. doi: 10.1038/s41597-022-01899-x 36596836 PMC9810617

[B24] Komorowski M. Green A. Tatham K. C. Seymour C. Antcliffe D. (2022). Sepsis biomarkers and diagnostic tools with a focus on machine learning. EBioMedicine 86, 104394. doi: 10.1016/j.ebiom.2022.104394 36470834 PMC9783125

[B25] Kurtz P. van den Boogaard M. Girard T. D. Hermann B. (2024). Acute encephalopathy in the ICU: a practical approach. Curr. Opin. Crit. Care 30, 106–120. doi: 10.1097/MCC.0000000000001144 38441156

[B26] Leung G. Middleton E. A. (2024). The role of platelets and megakaryocytes in sepsis and ARDS. J. Physiol. (Lond.) 602, 6047–6063. doi: 10.1113/JP284879 39425883

[B27] Li C. Zhao K. Ren Q. Chen L. Zhang Y. Wang G.. (2024). Development and validation of a model for predicting in-hospital mortality in patients with sepsis-associated kidney injury receiving renal replacement therapy: a retrospective cohort study based on the MIMIC-IV database. Front. Cell Infect. Microbiol 14. doi: 10.3389/fcimb.2024.1488505 PMC11570588 39559702

[B28] Lin J. Gu C. Sun Z. Zhang S. Nie S. (2024). Machine learning-based model for predicting the occurrence and mortality of nonpulmonary sepsis-associated ARDS. Sci. Rep. 14, 28240. doi: 10.1038/s41598-024-79899-7 39548234 PMC11568264

[B29] Ljungström L. Andersson R. Jacobsson G. (2019). Incidences of community onset severe sepsis, Sepsis-3 sepsis, and bacteremia in Sweden - A prospective population-based study. PloS One 14, e0225700. doi: 10.1371/journal.pone.0225700 31805110 PMC6894792

[B30] Lu X. Kang H. Zhou D. Li Q. (2022). Prediction and risk assessment of sepsis-associated encephalopathy in ICU based on interpretab le machine learning. Sci. Rep. 12, 22621. doi: 10.1038/s41598-022-27134-6 36587113 PMC9805434

[B31] Manabe T. Heneka M. T. (2022). Cerebral dysfunctions caused by sepsis during ageing. Nat. Rev. Immunol. 22, 444–458. doi: 10.1038/s41577-021-00643-7 34764472 PMC8582341

[B32] Muzambi R. Bhaskaran K. Smeeth L. Brayne C. Chaturvedi N. Warren-Gash C. (2021). Assessment of common infections and incident dementia using UK primary and secondary care data: a historical cohort study. Lancet Healthy Longevity 2, e426–e435. doi: 10.1016/S2666-7568(21)00118-5 34240064 PMC8245326

[B33] Peng L. Peng C. Yang F. Wang J. Zuo W. Cheng C.. (2022). Machine learning approach for the prediction of 30-day mortality in patients with sepsis-associated encephalopathy. BMC Med. Res. Methodol 22, 183. doi: 10.1186/s12874-022-01664-z 35787248 PMC9252033

[B34] Prithula J. Islam K. R. Kumar J. Tan T. L. Reaz M. Rahman T.. (2024). A novel classical machine learning framework for early sepsis prediction using electronic health record data from ICU patients. Comput. Biol. Med. 184, 109284. doi: 10.1016/j.compbiomed.2024.109284 39579661

[B35] Qiu X. Lei Y. P. Zhou R. X. (2023). SIRS, SOFA, qSOFA, and NEWS in the diagnosis of sepsis and prediction of adverse outcomes: a systematic review and meta-analysis. Expert Rev. Anti Infect. Ther. 21, 891–900. doi: 10.1080/14787210.2023.2237192 37450490

[B36] Ren C. Yao R. Q. Zhang H. Feng Y. W. Yao Y. M. (2020). Sepsis-associated encephalopathy: a vicious cycle of immunosuppression. J. Neuroinflammation 17, 14. doi: 10.1186/s12974-020-1701-3 31924221 PMC6953314

[B37] Slessarev M. Mahmoud O. McIntyre C. W. Ellis C. G. (2020). Cerebral blood flow deviations in critically ill patients: potential insult contributing to ischemic and hyperemic injury. Front. Med. (Lausanne) 7. doi: 10.3389/fmed.2020.615318 PMC7854569 33553208

[B38] Sonneville R. Benghanem S. Jeantin L. de Montmollin E. Doman M. Gaudemer A.. (2023). The spectrum of sepsis-associated encephalopathy: a clinical perspective. Crit. Care (London England) 27, 386. doi: 10.1186/s13054-023-04655-8 PMC10552444 37798769

[B39] Sonneville R. de Montmollin E. Poujade J. Garrouste-Orgeas M. Souweine B. Darmon M.. (2017). Potentially modifiable factors contributing to sepsis-associated encephalopathy. Intensive Care Med. 43, 1075–1084. doi: 10.1007/s00134-017-4807-z 28466149

[B40] Upadhyaya P. Wang J. Mathew D. T. Ali A. Tallowin S. Gann E.. (2025). Predicting sepsis induced hypotension patient attributes for restrictive vs liberal fluid strategy. Shock 63, 309–405. doi: 10.1097/SHK.0000000000002506 39617414

[B41] Wang S. Tang C. Liu Y. Border J. J. Roman R. J. Fan F. (2022). Impact of impaired cerebral blood flow autoregulation on cognitive impairment. Front. Aging 3. doi: 10.3389/fragi.2022.1077302 PMC9755178 36531742

[B42] Wang K. Tian J. Zheng C. Yang H. Ren J. Liu Y.. (2021). Interpretab le prediction of 3-year all-cause mortality in patients with heart failure caused by coronary heart disease based on machine learning and SHAP. Comput. Biol. Med. 137, 104813. doi: 10.1016/j.compbiomed.2021.104813 34481185

[B43] Yue S. Li S. Huang X. Liu J. Hou X. Zhao Y.. (2022). Machine learning for the prediction of acute kidney injury in patients with sepsis. J. Transl. Med. 20, 215. doi: 10.1186/s12967-022-03364-0 35562803 PMC9101823

[B44] Zhang Z. Guo L. Jia L. Duo H. Shen L. Zhao H. (2024). Factors contributing to sepsis-associated encephalopathy: a comprehensive systematic review and meta-analysis. Front. Med. (Lausanne) 11. doi: 10.3389/fmed.2024.1379019 PMC11148246 38835794

[B45] Zhang Y. Hu J. Hua T. Zhang J. Zhang Z. Yang M. (2023). Development of a machine learning-based prediction model for sepsis-associated delirium in the intensive care unit. Sci. Rep. 13, 12697. doi: 10.1038/s41598-023-38650-4 37542106 PMC10403605

