# Supplementary material for: Early prediction of sepsis associated encephalopathy in elderly ICU patients using machine learning models: a retrospective study based on the MIMIC-IV database
Source: Front Cell Infect Microbiol. 2025 Apr 17;15:1545979. doi: 10.3389/fcimb.2025.1545979 (PMC12043699; doi:10.3389/fcimb.2025.1545979)
Supplement: Supplementary file 1 [file Table1.docx]

**Supplementary Table1.** **Distribution of Missing Data.**

| Variable Names | Missing Data(%) |
| --- | --- |
| Heart rate (beats/minute) | 1.0 |
| Respiratory rate (beats/minute) | 0.1 |
| WBC(109/L） | 0.4 |
| Hemoglobin（g/dL） | 0.3 |
| PLT(109/L） | 0.5 |
| RBC(109/L） | 0.3 |
| RDW(%) | 0.4 |
| Hematocrit(%) | 0.5 |
| INR | 3.5 |
| PCO2(mmHg) | 14.0 |
| PTT (seconds) | 4.1 |
| PO2(mmHg) | 13.9 |
| PH | 12.6 |
| Lactate(mmol/l) | 13.5 |
| PT (seconds) | 3.5 |
