# Supplementary material for: Early prediction of sepsis associated encephalopathy in elderly ICU patients using machine learning models: a retrospective study based on the MIMIC-IV database
Source: Front Cell Infect Microbiol. 2025 Apr 17;15:1545979. doi: 10.3389/fcimb.2025.1545979 (PMC12043699; doi:10.3389/fcimb.2025.1545979)
Supplement: Supplementary file 2 [file Table2.docx]

**Supplementary Table2.The regression coefficients of the variables in the Lasso regression.**

| Variable | Lamda |
| --- | --- |
| Age | -0.00876421111014986 |
| Gender | 0 |
| Race | 0 |
| MAP | 0.0102331350925326 |
| RR | 0.00596200888912636 |
| HR | -0.0101947839085508 |
| SPO2 | -0.0179631853834377 |
| HLD | -0.217466540266649 |
| T2DM | -0.0944637905428851 |
| HTN | 0.279106837672536 |
| COPD | 0.153142102030251 |
| CKD | 0.0802744632353109 |
| IHD | -0.131919383402516 |
| T1DM | -0.15973654345609 |
| SOFA | 0.0292225944478406 |
| Oasis | 0.0508710023696425 |
| SapsⅡ | 0.0049267238753045 |
| Charlson | 0.0813863291800283 |
| ApsⅢ | -0.00452564816604652 |
| WBC | 0.0109745959881224 |
| Sodium | 0.0477780668106647 |
| Hemoglobin | -0.130319241318842 |
| Chloride | -0.024761841110453 |
| PLT | 0.00122244304569883 |
| RBC | -0.0928434530202784 |
| RDW | 0 |
| Potassium | -0.153312253984557 |
| Hematocrit | 0.0660200973179478 |
| Glucose | 0.00174880128067807 |
| Anion_gap | 0.0127964620072212 |
| INR | 0 |
| PCO_2_ | 0.00796868727946555 |
| PTT | 0.00226021692664768 |
| PO_2_ | -0.000811690026276421 |
| Creatinine | -0.137300329090975 |
| BUN | 0.00685595327453667 |
| Lactate | -0.0698799917611896 |
| PT | -0.00257218148232657 |
